# Alzheimer's disease diagnosis support for brain perfusion SPECT scans in a real-world clinical cohort

**DOI:** 10.1177/13872877251413790

**Published:** 2026-01-30

**Authors:** Sofia Michopoulou, Angus Prosser, Neil O’Brien, John Dickson, Matthew Guy, Jessica L. Teeling, Christopher M. Kipps

**Affiliations:** 1 Clinical and Experimental Sciences, Faculty of Medicine, University of Southampton, Southampton, UK; 2 University Hospital Southampton NHS Foundation Trust, Southampton, UK; 3 University College London Hospitals NHS Foundation Trust, London, UK; 4 School of Computer Science, University of Bristol, Bristol, UK; 5 Biological Sciences, University of Southampton, Southampton, UK

**Keywords:** Alzheimer's disease, artificial intelligence, brain perfusion, clinical decision support, real-world data, SPECT imaging

## Abstract

**Background:**

Dementia diagnosis is challenging and often delayed. Brain imaging techniques such as single-photon emission computed tomography (SPECT) imaging can help identify subtle changes in brain perfusion. Artificial intelligence methods may support results interpretation for early diagnosis.

**Objective:**

To develop and validate multivariate models for the early diagnosis of Alzheimer's disease (AD), using brain perfusion SPECT imaging and interpretable artificial intelligence methods in a real-world clinical setting.

**Methods:**

Two logistic regression models were developed using a training dataset of 420 SPECT scans and tested on an independent clinical dataset of 443 scans. Model 1 was designed to identify abnormal perfusion patterns, while Model 2 identified perfusion changes associated with AD. Input features were extracted from anatomical volumes of interest, with feature selection performed using the Minimum Redundancy Maximum Relevance (MRMR) algorithm.

**Results:**

The models demonstrated good classification performance using real-world clinical data. Model 1 achieved an area under receiver operator characteristic (AUROC) Curve of 0.89 (Sensitivity 76%, Specificity 87%) in identifying abnormal brain perfusion. Model 2 achieved an AUROC of 0.86 (Sensitivity 87%, Specificity 72%) in identifying AD.

**Conclusions:**

Multivariate logistic regression models trained on real-world clinical data show promise as clinical decision support tools for the diagnosis of AD from brain perfusion SPECT imaging. The models use features from clinically relevant brain regions, which enhances interpretability. Future research should focus on expanding model applicability to other dementia types and on prospective evaluation of their utility in improving diagnostic accuracy, consistency, and care pathways in diverse clinical environments.

## Introduction

Dementia diagnosis is a complex and prolonged process.^1,2^ Currently it is estimated that only about two thirds of patients living with dementia in the UK have a diagnosis, and that it takes upwards of 2 years on average from symptom onset to diagnosis.^2,3^ Dementia diagnosis is inherently subjective, and its accuracy depends on the clinician's experience.^1,4^ In Alzheimer's disease (AD), nearly a quarter of patients are misdiagnosed, with a large autopsy study indicating that the false negative and false positive rates for AD are 11.9% and 12.1% respectively.^5^

Early diagnosis and identification of dementia in individuals with cognitive complaints enables timely intervention and medical management, helping to preserve cognition, daily functioning, and quality of life for longer.^6^ It also reduces healthcare and residential care costs associated with dementia, and gives caregivers more time to adapt to their role, feel more competent to care for the patient, experience fewer psychological problems, and delay patient institutionalisation.^6,7^  Finally, early diagnosis is essential to the success of new disease-modifying therapies.^8–10^ Assessing the efficacy of new therapeutic methods requires not only early but also accurate diagnosis that is sensitive to small changes.^4^  

There is a clear need for objective methods for dementia diagnosis. Early dementia diagnosis is difficult, as diagnostic accuracy is lower in the early and pre-symptomatic stages.^11^ Mild cognitive impairment (MCI) precedes different types of dementia and impacts 1 in 5 people over the age of 65. From these, approximately 1 in 3 will convert to dementia; the challenge is to identify which patients will convert at the earliest possible stage.^12^

In emerging diagnostic paradigms using biomarkers, it might be thought that functional imaging markers are no longer needed. However, it is important to note that biomarker presence (particularly with pTau217) in plasma does not correlate with neuronal injury but rather the presence of amyloid. There remains controversy regarding the diagnostic framework definition for actual disease (international working group^13^ versus NIA-AA biological criteria^14^), and it remains likely that despite amyloid presence, a proportion of people will not develop meaningful clinical disease in their lifetime. It remains, therefore that markers of neuronal injury remain important; and that a hybrid approach to diagnosis incorporating both biomarker evidence of amyloid presence and additional evidence of neuronal injury are necessary for confirmed diagnosis of AD.

Functional neuroimaging techniques, including positron emission tomography (PET) and single photon emission computer tomography (SPECT) are key to evaluating neuronal injury and support early dementia diagnosis as they can identify subtle changes in brain metabolism and perfusion.^15^ While FDG PET provides higher spatial resolution, brain perfusion SPECT offers greater accessibility and lower cost, making it a useful option for resource-limited settings.^16^ A survey of imaging departments in the United Kingdom identified that FDG PET and perfusion SPECT are used in a similar number of centers, with the volume of SPECT scans being higher.^17^ However, the relative use of these modalities varies internationally. In countries where FDG PET is widely available, this is preferred due to its superior spatial resolution and stronger validation. Where perfusion SPECT remains in use, this often reflects differences in healthcare infrastructure, reimbursement policies, and scanner availability rather than diagnostic equivalence.^16,17^ The functional patterns of reduction in brain perfusion measured by SPECT help identify conversion from MCI to AD.^18^ 

Blood-based biomarkers, such as plasma neurofilament light, have emerged as scalable indicators of neuronal injury and are now incorporated into reviewed biomarker frameworks.^19^ However, these markers lack the regional specificity provided by functional imaging. FDG Pet and Perfusion SPECT enable localization of hypometabolism/hypoperfusion to clinically relevant brain regions, supporting differential diagnosis and complementing both fluid biomarkers and cognitive assessment.

A Cochrane review on functional neuroimaging highlighted the large variability in diagnostic performance in clinical practice.^1^ Clinical reporting of neuroimaging data primarily relies on visual assessment, which is subjective and dependent on the reader's experience.^4^ Quantitative analysis of neuroimaging data and the introduction of artificial intelligence (AI) methods for diagnosis support can help improve diagnostic accuracy and reduce variability between clinicians with different levels of experience in their interpretation of imaging data.^1,4,20–22^

Currently, most AI systems for dementia diagnosis have been trained and tested on data from the Alzheimer's Disease Neuroimaging Initiative (ADNI).^22^ This is a good choice for researchers since ADNI is large dataset of publicly available longitudinal data, collected using well-controlled protocols.^23^ It provides complete datasets for analysis and importantly facilitates comparison between different AI systems. However, generalization to clinical practice is limited and the accuracy of algorithms trained on ADNI often diminishes when they are applied to clinical datasets.^22^ For example, a study by Kloppel et al., applied a support vector machine classifier trained on ADNI with area under the receiver operating characteristic curve (AUROC) of 0.96 to data from a memory clinic and found markedly reduced accuracy in the clinical setting with an AUROC of 0.76 compared to the ADNI training dataset.^24^ For AI methods to be applicable in clinical practice, they would need to be robust for the variable quality of clinically acquired datasets and adaptable to heterogeneous cohorts commonly seen in clinical practice that include patients with pathologies other than AD as well as mixed pathologies.^21,22^

In this study we developed a multivariate model for the diagnosis of AD from brain perfusion SPECT scans, using training data from a real-world cohort to account for clinical heterogeneity. The model was validated using a prospectively acquired clinical dataset to evaluate applicability in a hospital setting. The model is intended for use as a support tool for clinical experts responsible for reporting brain perfusion scans. While not intended to confirm underlying pathology or guide therapy selection, the model aims to enhance the consistency and confidence of SPECT interpretation supporting current workflows where this modality is already in clinical use.

## Methods

### Participants and data

Data from participants referred to the Wessex Cognitive Disorders Clinic at University Hospital Southampton NHS Foundation Trust due to cognitive complaints were analyzed. Participants underwent a brain perfusion SPECT scan and provided written informed consent to share their data as part of the Brain Imaging In Dementia (BraIID) study [Ethics Approval Provided by NRES South Central - Hampshire A (15/SC/0231)]. Inclusion criteria were (1) referral for cognitive complaints, (2) availability of SPECT scan. No exclusion criteria were applied as the aim was to evaluate the use of brain SPECT in a real-world setting. SPECT data from 420 participants were used for training the decision support models.

### SPECT scan parameters

Participants were administered 500 MBq of Tc-99 m HMPAO in a quiet room with low lighting. The SPECT scan started 15 min after the radiopharmaceutical injection using the following imaging parameters: low energy high resolution collimators, circular orbit with the radius minimized for each patient, 128 projections at 25 s/projection, energy window center 140 keV, window width 15%, matrix 128 × 128, zoom 1.45.

Two different gamma cameras were used across the study period: an Infinia Hawkeye (GE Healthcare) until 2017, and an Intevo Bold (Siemens Healthineers) from 2018 onwards. To ensure consistency of quantitative image analysis across systems, images from both scanners were cross-verified using 3D printed phantoms with ink-printed perfusion patterns. These phantoms reproduced known patterns of tracer uptake and were used to verify alignment in image contrast, resolution, and count normalization between devices.

### Image processing and feature extraction

The SPECT scans were pre-processed using Statistical Parametric Mapping 12 (SPM12).^25,26^ Each scan was registered to the Montreal Neurological Institute (MNI) template. and smoothed using a 16 mm Gaussian filter. While this level of smoothing can obscure subtle focal differences, particularly in small cortical areas, it represents a deliberate trade-off. Brain perfusion SPECT has inherently low spatial resolution, and smoothing enhances signal-to-noise ratio and compensates for residual inter-subject anatomical variability. This approach improves the overall robustness of quantitative analysis despite loss of spatial precision. Count normalization was performed using the cerebellum as the reference region, with an age correction applied based on a mono-exponential fit of cerebellar counts derived from a database of healthy controls.

The input parameters used for training the model represent the SPECT counts in anatomical volumes of interest (VOIs) defined by the Automated Anatomical Labelling Atlas 3 (AAL3)^27^ averaged over the left and right hemispheres. Left-right averaging was applied to improve the signal-to-noise ratio and manage dimensionality by reducing the overall number of features for model training, with the aim of balancing signal optimization and model robustness whilst accepting possible loss of sensitivity to lateralized perfusion abnormalities.

### Feature selection and classification

Two multivariable logistic regression models were trained. Model 1 identified scan abnormality. Model 2 identified the presence of AD, including cases where AD was diagnosed alongside other dementias. This reflects the real-world diagnostic context in which AD frequently presents with mixed pathologies and recognizes that perfusion SPECT cannot confirm AD pathology in isolation. Specifically, the diagnosis of AD was derived from the neurologist's clinical report, which incorporated all available diagnostic information, including patient history, cognitive profile, and quantified perfusion SPECT imaging. SPECT findings were interpreted using statistical parametric mapping (SPM) to provide voxel-wise assessment of hypoperfusion relative to a normative database. This approach reflects the current recommendations of the National Institute for Health and Care Excellence, where perfusion SPECT or FDG PET is routinely used to support diagnostic formulation when structural imaging is inconclusive or equivocal.^28^ The two classification models were developed and applied independently. There was no hierarchical application, and both models were applied to all scans in the test dataset regardless of Model 1 outcome.

The Minimum Redundancy Maximum Relevance (MRMR) algorithm was used to select an optimal set of input parameters for model training by identifying features that are both highly relevant to the target variable and minimally redundant with one another.^29^ This approach is particularly well suited to medical datasets with high dimensionality and limited sample sizes, as it enhances model robustness and interpretability by reducing feature redundancy while preserving predictive information. To reduce overfitting risk, we implemented five-fold cross-validation within the training set during model development. Feature selection using the MRMR algorithm was performed within each fold, and the best-performing feature set was retained for final model training. Classification outcomes were reported using the accuracy, sensitivity, specificity and AUROC. These metrics were computed on an independent test dataset, which was prospectively acquired and not involved in model training or feature selection. To establish a performance baseline, naïve classifier accuracy was computed for both classification tasks by assigning the majority class to all test cases. Data analysis and results presentation used Matlab R2024b and SPSS v28.

## Results

### Participants and data

The logistic regression models were trained on 420 patients’ datasets, from which 318 had abnormal scans, and 210 had AD. The model's training performance on the 420 datasets was accessed using 5-fold cross validation and results are presented in Supplemental Table 1. Following training the models were tested on an independent test dataset of 443 patients, out of which 320 had abnormal scans, and 183 had AD.

### Selected input features

For Model 1, the Minimum Redundancy Maximum Relevance (MRMR) algorithm selected the supramarginal gyrus, medial temporal gyrus, lingual gyrus, supplementary motor area, and caudate nucleus as the most informative features for identifying perfusion abnormality. These regions are anatomically distributed across the cerebral cortex of the parietal, temporal, occipital, and frontal lobes as well as the subcortical basal ganglia. This spatial spread reflects the capacity of the model to detect heterogeneous patterns of hypoperfusion, which may arise from a range of underlying neurodegenerative processes. This is consistent with the varied presentation of cognitive syndromes encountered in this real-world clinical cohort, where multiple types of dementia, including AD, vascular dementia, and frontotemporal degeneration, result in distinct yet overlapping perfusion patterns.

For Model 2 the precuneus region was selected by MRMR as the single most descriptive feature for AD. There was no improvement in classification performance by including additional features in the logistic regression model for AD.

For all features, reduction in perfusion was associated with increasing likelihood of abnormality.

### Classification results for abnormal scans

**Figure 1.**
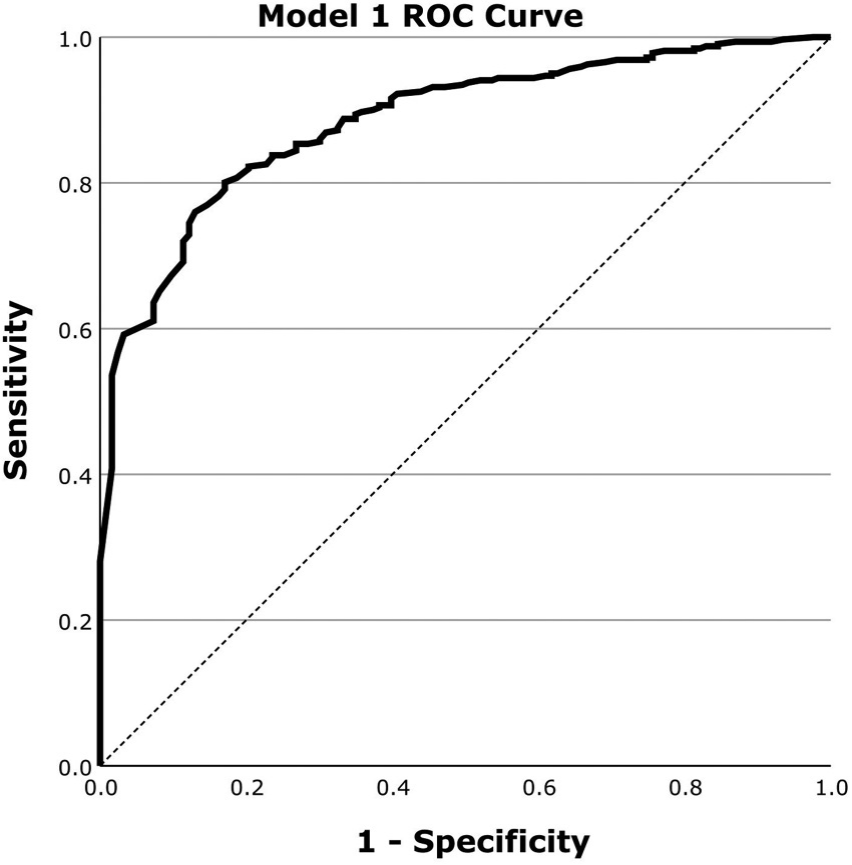
ROC curve for the evaluation of Model 1 for the classification of abnormal scans on a dataset of 443 scans using regional perfusion information from 5 regions selected by MRMR.

Figure 1 outlines the ROC curve for Model 1 evaluation in classifying abnormal scans. Table 1 outlines the corresponding AUROC, Accuracy, Sensitivity and Specificity for Model 1.

**Table 1.** Classification performance for Model 1: Abnormal Scan.

AUROC	Accuracy	Sensitivity	Specificity
0.89 [0.86–0.92]	79.1% [75.3%–82.9%]	76.0% [72.0%–80.0%]	87.0% [83.9%–90.1%]

Values expressed as Mean with [95% confidence interval].

### Classification results for Alzheimer's disease

**Figure 2.**
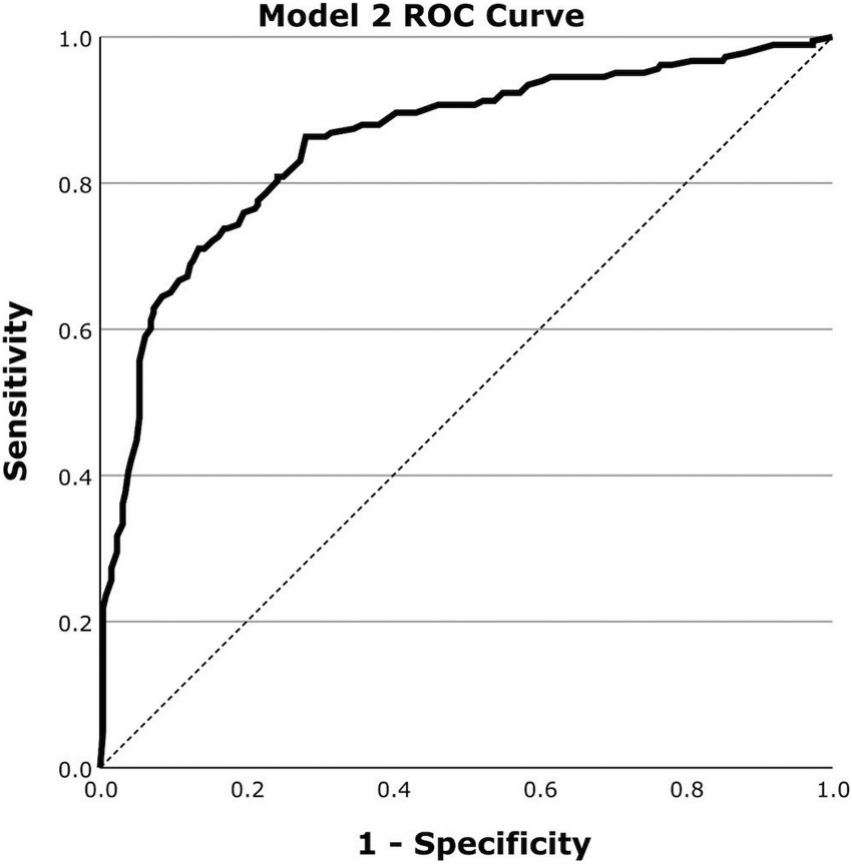
ROC curve for the evaluation of Model 2 for the classification of AD on a dataset of 443 scans using regional perfusion information from the precuneus region selected by MRMR.

Figure 2 outlines the ROC curve for Model 2 evaluation in classifying for AD. Table 2 outlines the corresponding AUROC, Accuracy, Sensitivity and Specificity for Model 2.

**Table 2.** Classification performance for Model 2: AD.

AUROC	Accuracy	Sensitivity	Specificity
0.86 [0.82, 0.89]	78.1% [74.2%, 82.0%]	86.9% [82.5%, 91.3%]	71.8% [66.5%, 77.1%]

Values expressed as Mean with [95% confidence interval].

### Interpretability of model outcomes

Interpretability of results is key, as the models were designed as decision support tools for clinical use. To facilitate interpretability, the regional perfusion features selected by the model were visually presented as bar graphs. Each bar depicts the z-score for a specific region, calculated by comparing the patient's regional SPECT count to the corresponding mean and standard deviation from a normal database of healthy controls. This standardized approach highlights deviations from typical perfusion patterns, allowing clinicians to easily identify which regions exhibit abnormal signal and contributed most strongly to the model's classification. By linking model decisions to anatomically meaningful and visually interpretable features, the output supports clinical reasoning and encourages user confidence in the AI-assisted diagnostic workflow.

**Figure 3.**
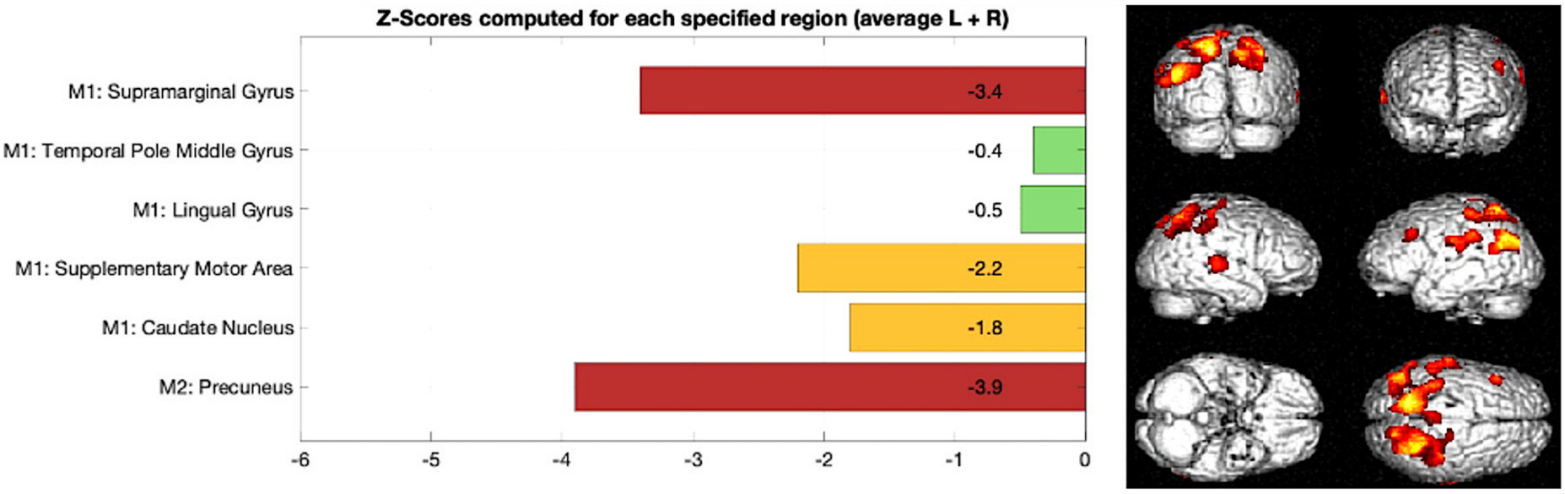
Example of AD, Left: z-score bar chart for regions used in Model 1 and Model 2 classification, Right: statistical parametric mapping results outlining clusters of significant reduction in brain perfusion in the parietal lobe compared to a normal database.

**Figure 4.**
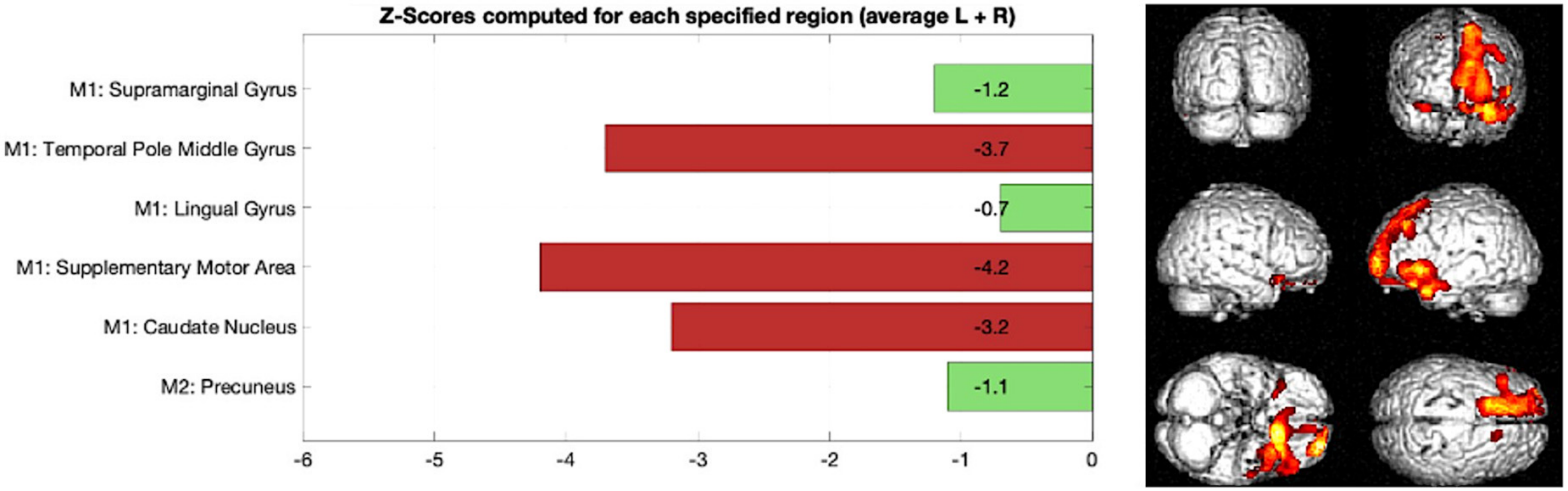
Example of frontotemporal dementia, Left: z-score bar chart for regions used in Model 1 and Model 2 classification, Right: statistical parametric mapping results outlining clusters of significant reduction in brain perfusion in frontal and temporal regions compared to a normal database.

Figure 3 outlines a z-score bar graph corresponding to a patient with AD and the associated statistical parametric mapping views of clusters of significant hypoperfusion to aid interpretation. Figure 4 illustrates an example for a patient with frontotemporal dementia.

Finally, the performance of naïve classifiers was calculated to provide a comparator for the two trained logistic regression models. Naïve classifiers provided an accuracy of 72.2% for Model 1 and 58.7% for Model 2.

## Discussion

This study demonstrates the effectiveness of two logistic regression models in classifying brain perfusion abnormalities and AD using SPECT imaging data. The overall classification accuracy for both models is promising, with AUROC values of 0.89 for abnormal scan classification (Model 1) and 0.86 for AD classification (Model 2).

While Model 1 yielded only a modest improvement over a naïve classifier due to the class imbalance and higher baseline accuracy of abnormal scan detection, Model 2 demonstrated a substantial gain, highlighting the added value of logistic regression modelling in distinguishing AD from other clinical presentations. The identification of the precuneus as the most predictive region for AD aligns with extensive literature on both FDG-PET and perfusion SPECT imaging. Posterior cingulate and precuneus hypometabolism is one of the earliest and most characteristic findings in FDG-PET of AD.^30^ Similarly, perfusion SPECT studies have consistently reported early and progressive hypoperfusion in the posterior parietal cortex, including the precuneus, in individuals with mild cognitive impairment and AD.^31,32^ This convergence across modalities supports the biological plausibility and face validity of our model's feature selection.

When compared to previously published work,^33,34^ the performance of our models aligns with known clinical outcomes and other SPECT image based diagnostic studies, suggesting potential utility in supporting diagnostic interpretation of perfusion SPECT scans. However, no formal inter-rater comparison was undertaken in this study, and such evaluation is warranted to further assess agreement with expert judgement. Focusing on AD detection, Model 2 demonstrates a sensitivity of 86.9% and specificity of 71.8%. This performance is close to SPECT based clinically reported sensitivity 71%-84% and specificity 70%-74%, outlining the potential of this Model for diagnosis support in a clinical setting.^33,34^

Our AD classification model achieved an AUROC of 0.86 in classifying patients with AD from patients with cognitive complaints using brain perfusion SPECT scans. This performance is lower than that reported by Ni et al., who applied a transfer learning approach using FDG PET data from the ADNI dataset to classify 247 perfusion SPECT scans, achieving an AUROC of 0.90.^35^ However, Ni et al.'s model focused solely on differentiating AD patients from healthy controls, which does not directly reflect the diagnostic challenges encountered in clinical memory clinics where the differential often involves patients presenting with cognitive complaints caused by a variety of pathologies.

A second study by Lien et al. tested the more relevant clinical scenario of classifying patients with AD from patients with mild cognitive impairment. They employed convolutional neural networks (ResNet architecture) to classify AD using SPECT images from 99 patients in a single-center cohort, reporting a maximum test accuracy of 68.8%.^36^ While their study explored deep learning in a relevant clinical dataset, the modest classification accuracy and absence of AUROC reporting limit direct comparability. Moreover, the comparatively lower performance may reflect differences in dataset size, diagnostic categories, and model optimization.

A key strength of this study is the use of a real-world clinical cohort, which supports meaningful integration into clinical diagnostic workflows. Unlike studies based on highly controlled datasets, such as those from ADNI), our approach captures the variability inherent in routine clinical practice. To maintain performance over time, future work should include recurring local validation to detect and adjust for potential distribution shifts that may affect model accuracy.^37^

Although the model demonstrated strong performance (AUROC 0.86–0.89), these results are based on single-center datasets with harmonized acquisition protocols. Generalizability to other clinical settings remains to be established. External validation is planned using data from a partner NHS hospital participating in the BRAIID study, with different scanning equipment and imaging protocols. We also aim to engage with international collaborators, including groups in Japan with large perfusion SPECT cohorts, to evaluate the model's robustness across diverse populations and scanning conditions.

In this study, no exclusion criteria were applied, in order to reflect real-world diagnostic complexity. We acknowledge that certain brain findings may influence the accuracy of predictive models. Specifically, within the BRAIID study cohort, 13% of participants had prior stroke, while no cases of brain tumors were identified. Future work should evaluate the impact of such prior conditions on classification accuracy.

The left-right averaging applied in this study may reduce sensitivity to lateralized perfusion patterns, which can be relevant in certain neurodegenerative presentations. This was a deliberate methodological choice to balance signal-to-noise optimization and dimensionality reduction given the dataset size constraints. This trade-off reflects our focus on developing a robust decision support tool for real-world use. Future work could develop additional models accounting for handedness.

A limitation of this study is that it focuses on AD without addressing other neurodegenerative conditions. AD accounts for 60–80% of all dementias, and further work is required to develop and validate models for less common forms of dementia, such as frontotemporal dementia (FTD).^38^ This will require the collection and analysis of larger datasets to ensure sufficient representation of dementias with low prevalence, required for models to accurately diagnose a broader range of neurodegenerative conditions.^22^ Specifically, in our dataset, patients with FTD accounted for 3.1% of our study population. Due to the low prevalence in the population, this dataset was insufficient to train and evaluate a dedicated logistic regression model specifically for FTD. However, in Figure 1 we presented an illustrative case in Figure 4 to demonstrate the ability of Model 1 to identify perfusion abnormalities in regions relevant to non-AD dementia. Expanding the analysis to include FTD is a priority identified with our patient and public involvement group. Ongoing efforts focus on increasing dataset size through access to data from multiple hospitals via the secure data environment, and on exploring alternative modelling approaches, such as nearest neighbor classifiers, which enable more robust parameter estimation in smaller datasets. A further limitation is the lack of pathological verification of amyloid plaques and tau tangles for gold standard diagnosis. As the reporting neurologist's diagnosis was used for training the model it should be acknowledged that this can be different to gold standard postmortem pathology results in up to 25% of cases.^39^ Additionally, deriving diagnostic labels from clinical reports which incorporate SPECT findings, creates an element of circularity, where the imaging modality being evaluated also contributes to the ground truth labels used for training. While this reflects real-world clinical decision-making, it can inflate performance estimates and underlines the importance of future validation against independent diagnostic standards, including pathological or longitudinal confirmation.

Furthermore, one should consider that mixed pathologies are common in clinically diagnosed AD, particularly in older adults; indeed, more than 50% of individuals with a clinical diagnosis of AD show additional neuropathological findings such as TDP-43, Lewy bodies, or cerebrovascular disease at autopsy.^40^ Our approach is therefore aligned with the realities of clinical practice, where such overlap is frequent and relevant to patient management.

Additionally, perfusion SPECT can be particularly informative for atypical AD, since symptoms typically follow the anatomical distribution of pathology rather than its molecular nature. Functional imaging demonstrates the neurobiological basis of atypical syndromes that fluid biomarkers, which lack all spatial resolution, cannot reveal. Moreover, SPECT assists in distinguishing structural and functional correlates of non-AD dementias and in highlighting mixed pathologies, which are common in older adults. These complementary strengths reinforce the continuing role of SPECT in supporting clinical diagnosis.

Another key consideration, beyond atypical AD, is the evolving role of biomarkers. Amyloid PET and emerging blood biomarkers (e.g., pTau217) are recognized as essential for confirming AD pathology, particularly in the context of anti-amyloid treatments. However, these biomarkers primarily indicate amyloid pathology and do not capture neuronal injury or explain symptomatic presentation. Functional imaging, in contrast, provides evidence of neuronal dysfunction and links symptoms to the anatomical distribution of pathology. A hybrid approach that combines biomarker evidence of amyloid with functional imaging evidence of neuronal injury is therefore likely to remain central to diagnostic confirmation and to guiding the use of disease-modifying therapies.

At the same time, access to these modalities remains limited in routine clinical practice, with adoption constrained by cost, infrastructure, and availability.^16,17,41^ Perfusion SPECT and FDG PET are more widely accessible and embedded in standard memory clinic workflows, particularly when structural imaging is inconclusive or when functional information is needed to support differential diagnosis.^28^ In the UK, perfusion SPECT is routinely used due to its greater accessibility compared to FDG PET. While its clinical use varies by region and is not universally adopted, FDG PET is increasingly preferred because it offers higher resolution imaging.

Building on the findings of this study, future work will focus on evaluating the clinical impact of AI-based diagnostic support tools, particularly their influence on diagnostic accuracy, consistency, and clinical decision-making, comparing scenarios with and without the use of AI assistance.^42,43^ It will be important to assess whether these tools enhance diagnostic confidence and reduce inter-observer variability, especially between experienced and less experienced clinicians. Further prospective studies will be required to evaluate how the use of AI models affects diagnostic agreement and downstream patient outcomes. The tool will be developed further using longitudinal data to assess the likelihood of progression to dementia for patients presenting with early MCI.

In conclusion, this study presented a diagnosis support tool for AD, using logistic regression models trained on brain perfusion SPECT imaging data. By leveraging real-world clinical data, the models demonstrated good classification accuracy, indicating their potential utility as decision support tools in clinical practice to enhance AD diagnosis and ultimately improve patient care. Future work will be directed towards broadening the scope of these models to non-AD dementias and assessing their impact in clinical decision making.

## Supplemental Material

sj-docx-1-alz-10.1177_13872877251413790 - Supplemental material for Alzheimer's disease diagnosis support for brain perfusion SPECT scans in a real-world clinical cohort Supplemental material, sj-docx-1-alz-10.1177_13872877251413790 for Alzheimer's disease diagnosis support for brain perfusion SPECT scans in a real-world clinical cohort by Sofia Michopoulou, Angus Prosser, Neil O’Brien, John Dickson, Matthew Guy, Jessica L. Teeling and Christopher M. Kipps in Journal of Alzheimer's Disease
