# Supplementary material for: Alzheimer's disease diagnosis support for brain perfusion SPECT scans in a real-world clinical cohort
Source: J Alzheimers Dis. 2026 Jan 30;110(1):192–200. doi: 10.1177/13872877251413790 (PMC12960786; doi:10.1177/13872877251413790)
Supplement: sj-docx-1-alz-10.1177_13872877251413790 - Supplemental material for Alzheimer's disease diagnosis support for brain perfusion SPECT scans in a real-world clinical cohort [file sj-docx-1-alz-10.1177_13872877251413790.docx]

**Supplemental Material**

**Alzheimer’s disease diagnosis support for brain perfusion SPECT scans in a real-world clinical cohort**

**Supplemental Table 1.** Classification performance for training set of 420 scans using 5-fold cross-validation

| Model | AUROC | Accuracy | Sensitivity | Specificity |
| --- | --- | --- | --- | --- |
| Model 1 | 0.93 | 88.6% | 70.6 | 94.3% |
| Model 2 | 0.86 | 76.2% | 78.1 | 74.3% |
